# Identifying the Shared Metabolite Biomarkers and Potential Intervention Targets for Multiple Sarcopenia-Related Phenotypes

**DOI:** 10.3390/ijms252212310

**Published:** 2024-11-16

**Authors:** Jia Luo, Jingxian Li, Weijing Wang, Ronghui Zhang, Dongfeng Zhang

**Affiliations:** Department of Epidemiology and Health Statistics, The School of Public Health of Qingdao University, Qingdao 266071, China; luojia9605@163.com (J.L.); jingxianli0715@163.com (J.L.); wangwj793@126.com (W.W.); zhangrh9634@163.com (R.Z.)

**Keywords:** sarcopenia, mendelian randomization, modifiable factors, colocalization analysis, metabolome

## Abstract

The relationship between circulating metabolites and sarcopenia-related phenotypes remains unclear. We explored the causality between circulating metabolites and sarcopenia-related phenotypes. Instrumental variables for the human metabolome were derived from the recently published GWAS, which included 690 plasma metabolites. Summary statistics for four sarcopenia phenotypes (whole-body lean mass (WBLM), usual walking pace, appendicular lean mass (ALM), and handgrip strength (HGS)) (both sexes, males and females) were obtained from relevant GWASs. We used MR to evaluate the association between circulating metabolites and sarcopenia-related phenotypes. Colocalization analysis was utilized to determine whether two associated signals were consistent with a shared causal variant rather than the confounding effect of linkage disequilibrium. Subsequently, we explored associations between modifiable risk factors and sarcopenia-related metabolites to explore which metabolites may serve as potential intervention targets through lifestyle modification. Genetically predicted plasma levels of 95 known metabolites were associated with sarcopenia-related phenotypes, and 27 metabolites were supported by robust evidence of colocalization, among which 13 metabolites had a cross-sarcopenia effect. These metabolites primarily included acyl carnitines, amino acids and their derivatives, and phospholipids. Specifically, our analyses supported causal relationships between 23, 6, and 15 metabolites and ALM, HGS, and WBLM, respectively. Seven relevant metabolites might be associated with six modifiable factors. We identified 27 metabolite biomarkers with robust causal evidence for sarcopenia-related phenotypes, highlighting 13 metabolites with a cross-sarcopenia effect, and prioritized several metabolites as the potential interventional targets of lifestyle changes. Our study provided new insight into the etiology and prevention of sarcopenia.

## 1. Introduction

Sarcopenia is characterized by a generalized and progressive skeletal muscle strength and mass decline and is primarily observed in the elderly [1]. Currently, most criteria have used combinations of muscle mass and strength as evaluation measures of sarcopenia [2,3,4]. The estimated prevalence of sarcopenia in populations of European ancestry is 23% for those older than 60 and 18% for those younger than 60, which also supports the idea that sarcopenia usually appears early in life [5]. Observational studies indicate that risk factors for sarcopenia include insufficient physical activity, malnutrition, smoking, and diabetes and that sarcopenia has also been linked to a range of negative health outcomes [6]. Therefore, the exploration of early biomarkers for sarcopenia holds the potential to advance the research on etiology, risk prediction, and precision prevention.

The human metabolome comprises products of metabolic processes, including intermediates and end products, which collectively present a metabolic fingerprint of an individual [7]. The development of omics-based technologies offers a window to unveil the complex mechanisms and pathological processes of diseases or phenotypes [8,9]. Specifically, metabolomics can reveal changes in intermediate metabolites or metabolic pathways to find new insights into diseases. Abnormal metabolism has been implicated in various diseases [10,11]. Evidence from observational metabolomics studies with candidate approaches supports an association between some metabolites and sarcopenia-related phenotypes [12,13,14]. However, previous studies based on cross-sectional design restrict causal inference, despite evidence indicating that broader metabolic disturbances occur in wasting. Additionally, most studies are limited to candidate metabolites, restricting a comprehensive understanding of the relationship between metabolites and sarcopenia. Recent studies also implied causal associations of several metabolites with sarcopenia, such as isovalerylcarnitine, docosapentaenoate, glycine, 1-arachidonoylglycerophosphocholine, pentadecanoate, 3-dehydrocarnitine, and epiandrosterone sulfate [15,16,17]. However, there is a lack of insight into the specific and shared metabolic markers between sarcopenia-related phenotypes, and sex differences in the association of metabolites with sarcopenia are not well defined.

Recent large-scale GWASs (1091 metabolites, 690 of which had available mQTL) have significantly advanced our knowledge of the genetic architecture underlying the human metabolome [18]. Meanwhile, the availability of muscle strength and mass phenotypes (HGS, ALM, WBLM, and usual walking pace) with associated genotypes provides a valuable resource for evaluating causal association by MR. MR employs genetic variants as proxies to achieve robust causal inference between a given exposure and the outcome with minimal reverse causation or confounding effects [19]. Therefore, we conducted a metabolome-wide MR analysis to assess the causal association between metabolites and sarcopenia-related phenotypes and to explore the cross-sarcopenia effects of metabolites. Subsequently, we further investigate the association across different genders.

## 2. Results

### 2.1. Metabolome-Wide MR Identified 118 Sarcopenia-Related Metabolites

The reporting of this MR study followed the STROBE-MR checklist (Supplementary File S1). The data sources and detailed information are presented in Table S1. The F-statistic for the genetic instruments was larger than the normally selected value of 10, suggesting no weak instrumental variable (Table S2). The number of nominally significant metabolites (*p* < 0.05) across the sarcopenia traits ranged from 239 for ALM to 73 for usual walking pace (Table 1). There were 118 metabolites (95 known and 23 unknown metabolites) significantly associated with four phenotypes after multiple testing corrections, including 78 known metabolites for ALM, 11 known metabolites for HGS, 47 known metabolites for WBLM, and 1 known metabolite for usual walking pace (Tables S4–S7). Among these known metabolites, no significant pleiotropy or heterogeneity was observed (Table S8).

In the sex-stratified analyses, we observed significant associations between 50, 19, 3, and 1 known metabolite and ALM, WBLM, HGS, and usual walking pace in males, respectively (Table 1 and Tables S9–S12). In females, we observed significant associations between 64, 23, and 8 known metabolites and ALM, WBLM, and HGS, respectively (Table 1 and Tables S13–S16). For ALM, HGS, and WBLM, 34, 13, and 6 known metabolites were significant in both the male and female stratified analyses, respectively. There were 16 and 6 known metabolites that were significantly associated with ALM and WBLM only in males. For ALM, WBLM, and HGS, 30, 10, and 5 known metabolites were significant only in females, respectively.

Among the 11 known metabolites significantly associated with HGS, 8 metabolites were subsequently validated. The genetically determined levels of myristoylcarnitine, arachidoylcarnitine, S-adenosylhomocysteine (SAH), and 3-hydroxyoleoylcarnitine were positively related to the risk of HGS weakness [OR (95 CI): 1.26 (1.11–1.43), 1.19 (1.07–1.34), 1.15 (1.06–1.25), and 1.20 (1.07–1.36), respectively. The genetically determined levels of four metabolites (glycine, isovalerylglycine, cinnamoylglycine, and gamma-glutamylglycine) were negatively correlated with the risk of HGS weakness (Table S17).

### 2.2. Colocalization Analysis Supports 27 Known Metabolites

**Figure 1:**
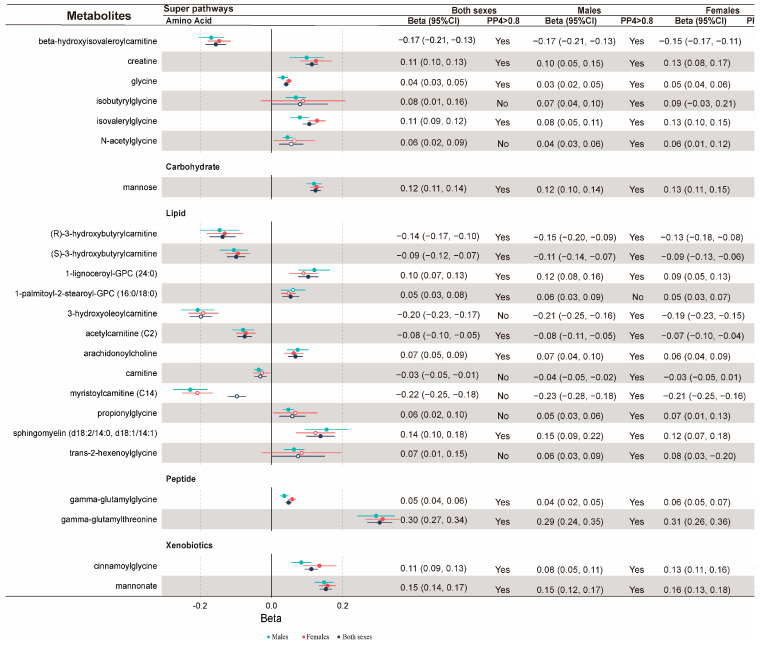
ALM-related metabolites from MR with robust colocalization evidence. Light blue represents males, red represents females, and black represents the overall population. The dots indicate the effect sizes of the corresponding metabolites, with horizontal lines showing the corresponding 95% confidence intervals (CIs). Solid dots indicate evidence supported by colocalization, while hollow dots indicate a lack of colocalization evidence.

**Figure 2:**
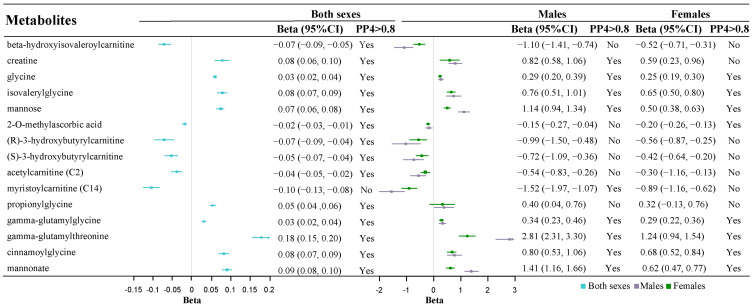
WBLM-related metabolites from MR with robust colocalization evidence. Light blue represents the overall population, light purple represents males, and green represents females. Squares indicate the effect sizes of the corresponding metabolites, with horizontal lines representing 95% confidence intervals (CIs).

**Figure 3:**
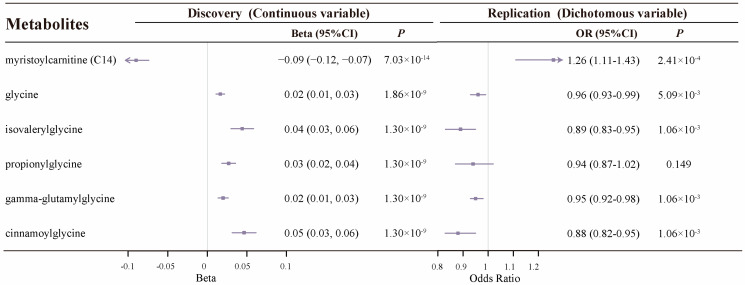
HGS-related metabolites from MR with robust colocalization evidence and results of replication analysis. Squares indicate the effect sizes of the corresponding metabolites, with horizontal lines representing the 95% confidence intervals (CIs).

Among the 95 known metabolites associated with sarcopenia-related phenotypes, 27 metabolites had strong colocalization evidence (PP_4_ > 0.80) under different windows (±250 kb or ±500 kb) and priors (P_12_ = 1 × 10^−5^ or P_12_ = 1 × 10^−6^), suggesting the high probability of a shared causal variant between metabolites and sarcopenia-related phenotypes (Table S18). Specifically, there is significant colocalization support for the associations of 23, 6, and 15 known metabolites with ALM (Figure 1), HGS (Figure 2), and WBLM (Figure 3), respectively.

Totals of 23 and 13 metabolites were significantly associated with at least one sarcopenia-related phenotype in males and females, respectively, with colocalization evidence supports. Among them, 12 metabolites were significant in both sexes, primarily falling into the amino acids and their derivatives, categories of carnitines and their derivatives, and carbohydrate metabolites. The male-specific sarcopenia-related metabolites included myristoylcarnitine, propionylglycine, sphingomyelin, isobutyrylglycine, arachidoylcarnitine, 1-lignoceroyl-GPC (24:0), N-acetylglycine, carnitine, arachidonoylcholine, trans-2-hexenoylglycine, and 3-hydroxyoleoylcarnitine, while the only female-specific sarcopenia-related metabolite was 2-O-methylascorbic acid.

### 2.3. Thirteen Metabolites with Robust Colocalization Evidence Have Cross-Sarcopenia Effect

Among 27 identified sarcopenia-related metabolites, 13 metabolites showed cross-sarcopenia effects. Specifically, the genetically determined plasma myristoylcarnitine level was related to lower ALM, WBLM, and HGS. The higher levels of five other metabolites (glycine, isovalerylglycine, propionylglycine, gamma-glutamylglycine, and cinnamoylglycine) were associated with higher ALM, WBLM, and HGS. The higher levels of mannose, creatine, and mannonate were associated with higher ALM and WBLM. The lower levels of four other metabolites (beta-hydroxyisovaleroylcarnitine, (R)-3-hydroxybutyrylcarnitine, (S)-hydroxybutyrylcarnitine, and acetylcarnitine) were associated with higher ALM and WBLM (Table 2).

### 2.4. Metabolic Pathway Analysis

Metabolic pathway analysis was performed among the metabolites with robust colocalization evidence using Metaconflict 5.0. A total of 12 metabolic pathways were detected from two databases, including 7 from both the KEGG and the Small Molecule Pathway databases, as well as 5 from the KEGG dataset. Four important metabolic pathways potentially involved in muscle mass were identified in the metabolic pathway analysis (Table S19). The results indicated that the “glycerophospholipid metabolism”, “glycine, serine and threonine metabolism”, “linoleic acid metabolism”, and “alpha-Linolenic acid metabolism” pathways might be relevant to ALM and WBLM (*p* < 0.05). We did not find pathways associated with HGS and usual walking pace due to limited metabolites.

### 2.5. Six Potential Modifiable Factors Associated with Sarcopenia-Related Metabolites

In the analysis of the associations of 38 modifiable factors with 27 sarcopenia-related metabolites, 6 modifiable factors (1 obesity-related factor: WHRadjBMI; 3 lifestyle factors: television watching, sleep duration, and smoking initiation; and 2 dietary factors: tea consumption and milk intake) were significantly associated with 7 metabolites. Among 13 metabolites with cross-sarcopenia effects, beta-hydroxyisovaleroylcarnitine was positively associated with smoking initiation and short sleep duration, while gamma-glutamylglycine, glycine, and mannose were positively related to milk intake and lower WHRadjBMI (Table 3).

## 3. Discussion

In this study, we systematically investigated the causal association between 690 blood metabolites and sarcopenia-related phenotypes in both sexes, male and female, respectively. We identified 95 known metabolites associated with sarcopenia-related phenotypes, with the majority showing significance in sex-stratified analyses. A total of 27 metabolites were prioritized after Bayesian colocalization analysis, and 13 of these had a cross-sarcopenia effect. Additionally, 23 metabolites in the males and 13 in the females were associated with at least one sarcopenia phenotype, supported by strong colocalization evidence. Of these, 12 metabolites were same in the males and females.

Glycine has been found to have potential effects on muscle health. Insufficient synthesis of glycine is associated with muscle weakness [20,21]. A glycine supplement can protect muscles from the impact of various diseases, including cachexia [22,23,24]. Gamma-glutamylglycine and gamma-glutamylthreonine were observed to be associated with muscle mass and strength. It can be resynthesized into glutathione under the catalytic action of gamma-glutamyl transpeptidase [8]. Research suggests that glutathione may help maintain muscle health through antioxidants [25]. Isovalerylglycine is commonly used for the screening of isovaleric acidemia in newborns. However, a previous study identified it as a nutritional and health-related biomarker for diagnosing or detecting resistance to diet-induced obesity [26]. A higher isovalerylglycine level indicates an increased likelihood that obesity induced by a high-fat diet can be resisted [26]. Cinnamoylglycine is typically formed through the metabolism of dietary polyphenols by gut microbiota, suggesting a potential role of dietary polyphenols or gut microbiota in maintaining muscle health [27]. Contrary to our findings, Lustgarten et al. found through principal component analysis that the principal component containing cinnamoylglycine was negatively correlated with muscle strength in adults [28,29]. The reason for this discrepancy may lie in the differences in research methodologies.

Our results indicated a causal effect of acyl carnitines on sarcopenia-related phenotypes. Acyl carnitines are predominantly derived from skeletal muscles and play a crucial role in intracellular fatty acid metabolism and transport. Acyl carnitines accumulation is observed in certain conditions, such as type 2 diabetes, cardiac ischemia, or inherited fatty acid oxidation disorders [30], emphasizing the impact of these conditions on muscle function. Previous studies suggested that long-chain acyl carnitines are implicated in activating insulin resistance and inflammatory responses in muscle tissues [30,31,32,33]. Under pathological conditions, such as fatty acid oxidation disorders or myocardial ischemia, acyl carnitines may induce oxidative stress in muscle cells [30,34]. In vitro experiments indicated that low concentrations of acyl carnitines may initiate Ca-dependent IL-6 responses by increasing Ca2+ in muscle cells, potentially resulting in muscle cell apoptosis [35]. These findings collectively emphasize the significant impact of acyl carnitines on muscle physiology. As one of the most popular sports supplements, creatine could enhance adenosine triphosphate (ATP) resynthesis in cells, thereby contributing to improved physical performance, including maximal strength, maximal work output, sprinting performance, and fat-free mass [36,37].

Our results found that sarcopenia-related metabolites were largely consistent across the sexes, though more sarcopenia-related metabolites were identified in the males than in the females. Although the underlying mechanisms remain unclear, we propose several possible explanations. First, sex differences in metabolite levels have been documented in prior studies, which show that males exhibit higher concentrations of metabolites, such as creatine and fatty acid oxidation-related products, including carnitines and acylcarnitine, compared to females [38,39]. These differences may arise from estrogen regulation [40], inflammatory responses, muscle mass and body composition, as well as endogenous metabolite synthesis [38], potentially contributing to the stronger associations observed between some metabolites and sarcopenia in males. Additionally, male-specific environmental factors, such as high-intensity training and higher basal metabolic rates, may influence the concentration or functional activity of certain amino acid metabolites [41], which may also account for the observed gender difference.

Several modifiable risk factors (WHRadjBMI, leisure television watching, sleep duration, smoking initiation, tea consumption, and milk intake) are associated with seven sarcopenia-related metabolites. Previous studies have also identified these factors as being associated with sarcopenia [42]. However, our hypothesis is based on genetic variations as proxies, which limits our ability to fully evaluate potential confounding factors and only provides potential clues. Rigorously designed intervention studies are needed in the future to assess whether changes in lifestyle behaviors could affect the sarcopenia risk through influence-identified metabolites.

This study has the following advantages: Firstly, we performed a comprehensive and systematic study to explore the causal association between plasma metabolites and the sarcopenia-related phenotypes, providing a comprehensive view with which to understand the etiological role of metabolites in muscle function. Secondly, the use of the MR and Bayesian colocalization methods reduced the impact of confounding factors and reverse causation, making the current study potentially more robust than observational studies. Thirdly, we identified modifiable factors of relevant metabolites, prioritizing potential interventional metabolite targets of lifestyle changes to treat sarcopenia.

However, our study has weaknesses that warrant caution. Firstly, the current study focused on sarcopenia-related phenotypes rather than sarcopenia diagnosed by internationally recognized criteria, such as those defined by the EWGSOP or AWGS. This is due to the current lack of GWASs on sarcopenia as defined by the above standards. Moreover, the sarcopenia-related phenotypes we selected are common indicators in the internationally recognized criteria and have been widely used in previous research [43]. Secondly, blood metabolites are commonly used for disease diagnosis or screening due to the accessibility and low invasiveness. Therefore, this study focuses on plasma metabolites, but evaluating the role of metabolites in other tissues, especially muscle tissue, could offer further perspectives on the pathology of sarcopenia. Thirdly, due to the current lack of GWASs on ALM, WBLM, and walking pace in other populations, we were only able to validate metabolites associated with handgrip strength. Our results will require further validation through experimental studies or population-based research in the future. Finally, since we used genetically determined metabolites to explore their association with sarcopenia-related phenotypes, we were unable to provide actual metabolite concentrations in sarcopenia patients. This limitation restricts the clinical applicability of our findings and calls for further research in the future.

## 4. Methods and Materials

### 4.1. Overall Study Design

**Figure 4:**
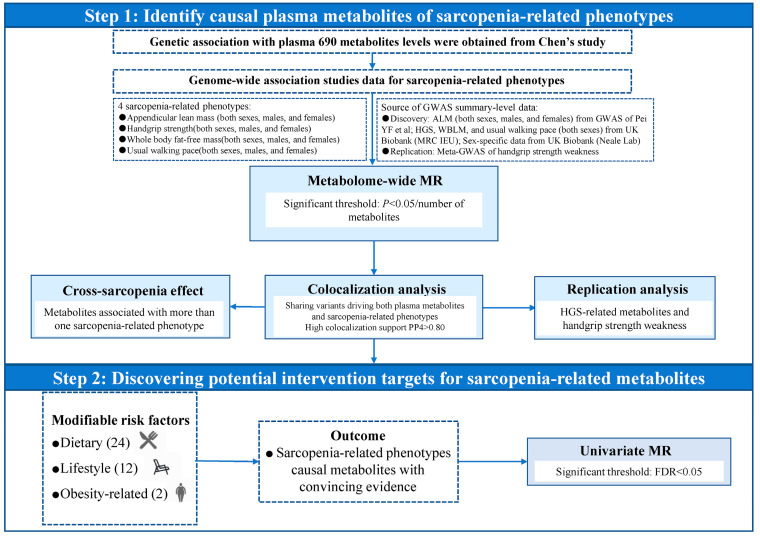
Overview of the study design and analytical process.

The study design is outlined in Figure 4. Initially, we performed a two-sample MR analysis using genetic variants from the recently published metabolomics study as instrumental variables to explore the association between metabolites and sarcopenia-related phenotypes. Subsequently, we conducted colocalization analysis to verify the causal relationships. Metabolic pathway analysis was further conducted to identify the underlying metabolite pathways or groups that might be related to the biological process of sarcopenia. Finally, another MR analysis was conducted between the modifiable risk factors and sarcopenia-related metabolites to identify which metabolites might serve as potential interventional targets of modifiable lifestyle factors.

### 4.2. Data Sources and Study Population

Instrumental variables for the human metabolome were derived from the recently published largest GWAS, which included 690 blood metabolites (571 known and 119 unknown) measured using the Metabolon HD4 platform among 8299 individuals of European ancestry [18]. The mean age was 62.4 ± 9.9 years. As in previous studies, we did not use sarcopenia directly defined by the EWGSOP criteria as an outcome; instead, we analyzed related indicators of sarcopenia [44]. We obtained summary statistics for single-nucleotide polymorphisms (SNPs) associated with sarcopenia-related phenotypes from previous GWASs [45,46]. In brief, ALM and WBLM were selected as measures of muscle mass; both of these are valid predictors of sarcopenia [47]. HGS and usual walking pace were selected as measures of muscle strength [48]. We used the full summary statistics data from a GWAS on ALM, which contained 450,243 participants of European ancestry, and ALM was measured using bioelectrical impedance analysis (BIA) [45]. For HGS, usual walking pace, and WBLM, full summary statistics data from UK Biobank (n = 461,089, 459,915 and 454,850, respectively; aged between 48 and 73 at recruitment) were used.

We also obtained sex-specific full summary statistics data on WBLM, HGS, and usual walking pace from UK Biobank (Neale Lab) in the sex-stratified analysis [49]. For the WBLM, the sample size was 163,815 for the males and 190,993 for the females. For HGS, the sample size was 166,424 for the males and 193,280 for the females. For usual walking pace, the sample size was 165,967 for the males and 193,007 for the females. The GWAS of ALM conducted by Pei et al. contains 205,513 males (the mean age was 57.0 ± 8.1 years) and 244,730 females (the mean age was 56.6 ± 7.9 years) [45]. The original publications obtained the ethics approvals from the relevant authorities, and informed consent was provided by all the participants.

### 4.3. Metabolome-Wide MR Analysis

In the present MR analysis, the following criteria were applied to choose the instrumental variables (IVs) of the plasma metabolites: (i) SNPs associated with a corresponding metabolite at the genome-wide significance level (*p* < 5 × 10^−8^); (ii) due to the complex linkage disequilibrium (LD) structure of SNPs within the human major histocompatibility complex region, SNPs within this region were excluded; (iii) to identify independent IVs for a given metabolite, the clumping process was used (r^2^ < 0.001); and (iv) the R^2^ and F-statistic were used to measure the strength of the IVs, where R^2^ was the variance explained by genetic variance, and an F-statistic of less than 10 was considered indicative of a weak IV [50]. Finally, 690 unique metabolites and 1291 IVs were included in the sequence analysis. Table S2 presents detailed information on the IVs.

The MR analysis utilized the “TwoSampleMR” R package [51]. If the metabolites had only a single IV, we utilized the Wald ratio method to evaluate the changes in ALM, WBLM, HGS, and usual walking pace for a per SD increase in plasma metabolites, as proxied by the IVs. For the metabolites with more than one genetic proxy, we used the inverse-variance weighted (IVW) method, and the heterogeneity of the IVs was estimated by a heterogeneity test based on the Q statistic. Additional analyses, including weighted median, simple mode, MR-Egger, and weighted mode, were also conducted to detect potential horizontal pleiotropy. Bonferroni correction was used for multiple testing and *p* < 7.25 × 10^−5^ (0.05/690) was considered as the significance threshold.

Due to the lack of available replication datasets for ALM, WBLM, and usual walking pace, the replication analysis only validated the causal relationship between HGS-related metabolites and HGS weakness. Summary statistics data of HGS weakness were obtained from a previous GWAS meta-analysis, where the definition of HGS weakness was in accordance with the European Working Group on Sarcopenia in Older People (EWGSOP) criteria (<20 kg for females; HGS < 30 kg for males) [52]. A false discovery rate (FDR) < 0.05 was considered as the significance threshold in the replication analysis.

### 4.4. Colocalization Analysis

The Bayesian colocalization analysis was conducted using the “coloc” R package to determinate whether two associated signals (muscle strength- and mass-related metabolites and their corresponding phenotypes) were consistent with a shared causal variant rather than the confounding effect of LD [53]. This analysis included five hypotheses: (H0) no causal variant for either the metabolites or phenotypes in the locus; (H1) one causal variant for a metabolite only; (H2) one causal variant for a muscle strength and mass phenotype only; (H3) two different causal variants for the metabolites and phenotype; and (H4) the metabolite and phenotype shared a common causal variant. For each metabolite, we performed colocalization analysis by including SNPs within a 500 kb window upstream and downstream of the instrumental variables, with a default parameter set at *P*_12_ = 1 × 10^−5^ (prior probability that an SNP is associated with both metabolites and sarcopenia-related phenotypes). Due to the sensitivity of colocalization analysis to the prior and window size, we assessed the robustness of the colocalization by using a smaller window size (±250 kb) and prior (*P*_12_ = 1 × 10^−6^) in additional analyses [54]. The posterior probability of H4 (PP4) being greater than 0.80 across different windows and priors was considered as strong colocalization evidence [55].

### 4.5. Metabolic Pathway Analysis

For the potential suitability of the sarcopenia causal metabolites with convincing evidence, metabolic pathway analysis was conducted using the web-based Metaconflict 5.0 to identify underlying metabolite groups or pathways which might be involved in the biological process of sarcopenia [56].

### 4.6. Associations Between Modifiable Risk Factors and Sarcopenia-Related Metabolites

To investigate the associations between potential modifiable lifestyle factors and sarcopenia-related metabolites with convincing colocalization evidence, we conducted additional univariate MR analyses. These analyses included a total of 38 modifiable risk factors, comprising 12 related to lifestyle behaviors, 24 related to diet, and 2 related to obesity (Table S3). FDR < 0.05 was defined as the significance level. R version 4.1.1 was used to conduct all the analyses.

## 5. Conclusions

In summary, we identified 27 plasma metabolite biomarkers with convincing evidence for sarcopenia-related phenotypes and highlighted 13 metabolites with a cross-sarcopenia effect, and we prioritized the potential intervention metabolite targets of lifestyle changes. Our study offered new clues into the prevention and etiology of sarcopenia. Future studies are needed to validate the findings and investigate whether the identified metabolites could serve as potential intervention targets through lifestyles or drugs to reduce the risk of sarcopenia.

## Figures and Tables

**Table 1:** Summary of results from metabolome-wide MR analysis of sarcopenia-related phenotypes.

Phenotypes	Stratified Analysis	Number of Metabolites		
		with Available IVs	*p* < 0.05	*p* < Bonferroni-Corrected Threshold
ALM	Both sexes	665	239	95
	Male	665	184	60
	Female	665	211	77
HGS	Both sexes	645	86	18
	Male	657	86	5
	Female	657	92	12
WBLM	Both sexes	645	198	64
	Male	657	142	36
	Female	657	123	31
Usual walking pace	Both sexes	645	73	2
	Male	657	25	2
	Female	657	49	0

IVs, instrumental variables; WBLM, whole-body lean mass; ALM, appendicular lean mass; HGS, handgrip strength.

**Table 2:** Results of metabolites with cross-sarcopenia effect in Mendelian randomization analysis.

Metabolites	Association Between Metabolites with Sarcopenia-Related Traits			
	Sarcopenia-Related Traits	SNP	Beta (95%CI)	*p* Value
myristoylcarnitine	ALM *	1	−0.23 (−0.28, −0.18)	1.6 × 10^−20^
	WBLM *	1	−1.52 (−1.97, −1.07)	2.91 × 10^−11^
	HGS	1	−0.09 (−0.12, −0.07)	7.03 × 10^−14^
glycine	ALM	2	0.04 (0.03, 0.05)	7.52 × 10^−29^
	WBLM	2	0.03 (0.02, 0.04)	4.38 × 10^−38^
	HGS	2	0.02 (0.01, 0.03)	1.86 × 10^−9^
isovalerylglycine	ALM *	1	0.04 (0.03, 0.06)	7.33 × 10^−9^
	WBLM	1	0.08 (0.07, 0.09)	6.02 × 10^−37^
	HGS	1	0.04 (0.03, 0.06)	1.3 × 10^−9^
propionylglycine	ALM *	2	0.05 (0.03, 0.06)	2.12 × 10^−9^
	WBLM	2	0.05 (0.04, 0.06)	6.02 × 10^−37^
	HGS	2	0.03 (0.02, 0.04)	1.3 × 10^−9^
gamma-glutamylglycine	ALM	1	0.30 (0.27, 0.34)	2.29 × 10^−31^
	WBLM	1	0.03 (0.02, 0.04)	6.02 × 10^−37^
	HGS	1	0.02 (0.01, 0.03)	1.3 × 10^−9^
cinnamoylglycine	ALM	1	0.08 (0.05, 0.11)	2.29 × 10^−31^
	WBLM	1	0.08 (0.07, 0.09)	6.02 × 10^−37^
	HGS	1	0.05 (0.03, 0.06)	1.30 × 10^−9^
mannose	ALM	1	0.12 (0.11, 0.14)	8.21 × 10^−65^
	WBLM	1	0.07 (0.06, 0.08)	1.1 × 10^−50^
creatine	ALM	2	0.11 (0.10, 0.13)	4.27 × 10^−39^
	WBLM	2	0.08 (0.06, 0.10)	3.39 × 10^−17^
mannonate	ALM	1	0.15 (0.14, 0.17)	8.21 × 10^−65^
	WBLM	1	0.09 (0.08, 0.10)	1.1 × 10^−50^
beta-hydroxyisovaleroylcarnitine	ALM	2	−0.17 (−0.21, −0.13)	7.65 × 10^−26^
	WBLM	2	−0.07 (−0.09, −0.05)	1.45 × 10^−18^
(R)-3-hydroxybutyrylcarnitine	ALM	1	−0.14 (−0.17, −0.10)	1.79 × 10^−13^
	WBLM	1	−0.07 (−0.09, −0.04)	1.48 × 10^−8^
(S)-3-hydroxybutyrylcarnitine	ALM	1	−0.09 (−0.12, −0.07)	4.51 × 10^−14^
	WBLM	1	−0.05 (−0.07, −0.04)	2.27 × 10^−9^
acetylcarnitine	ALM	1	−0.08 (−0.10, −0.05)	1.79 × 10^−13^
	WBLM	1	−0.04 (−0.05, −0.02)	1.48 × 10^−8^

* Colocalization evidence only supports associations of these metabolites with sarcopenia-related phenotypes in males.

**Table 3:** MR analysis of modifiable lifestyle factors with sarcopenia-related metabolites.

Metabolites	Modifiable Factors	Method	nSNP	Beta (95%CI)	*p*	FDR
1-lignoceroyl-GPC (24:0)	Television watching	IVW	88	−0.44 (−0.66, −0.22)	1.07 × 10^−4^	4.05 × 10^−3^
beta-hydroxyisovaleroylcarnitine	Sleep duration	IVW	58	−0.007 (−0.010, −003)	1.13 × 10^−3^	2.63 × 10^−2^
beta-hydroxyisovaleroylcarnitine	Smoking initiation	IVW	192	0.20 (0.08, 0.32)	1.38 × 10^−3^	2.63 × 10^−2^
gamma-glutamylglycine	WHRadjBMI	IVW	292	−0.21 (−0.32, −0.10)	2.68 × 10^−4^	1.02 × 10^−2^
glycine	WHRadjBMI	IVW	292	−0.23 (−0.35, −0.12)	3.89 × 10^−5^	1.48 × 10^−3^
levulinoylcarnitine	Tea consumption	IVW	12	1.82 (0.83, 2.81)	3.19 × 10^−4^	1.21 × 10^−2^
mannose	Smoking initiation	IVW	192	0.22 (0.10, 0.35)	2.98 × 10^−4^	1.13 × 10^−2^
mannose	Milk intake	Wald ratio	1	0.08 (0.03, 0.13)	2.41 × 10^−3^	4.59 × 10^−2^
sphingomyelin (d18:2/14:0, 18:1/14:1)	WHRadjBMI	IVW	292	−0.22 (−0.31, −0.12)	7.77 × 10^−6^	2.95 × 10^−4^

IVW, inverse-variance weighted; FDR, false discovery rate; WHRadjBMI, waist-to-hip ratio adjusted for body mass index.

## Data Availability

The findings of this study can be found in its supplementary materials. GWAS summary data of sarcopenia-related phenotypes are available at http://www.nealelab.is/uk-biobank (accessed on 6 September 2023) and https://gwas.mrcieu.ac.uk/ (accessed on 6 September 2023). The GWAS summary data of metabolites are available at GWAS Catalog (https://www.ebi.ac.uk/gwas/ (accessed on 6 September 2023)). The GWAS summary data of replication analysis are available from the original publications.

## Supplementary Materials

The following supporting information can be downloaded at: https://www.mdpi.com/article/10.3390/ijms252212310/s1.
