# Supplementary material for: Identifying the Shared Metabolite Biomarkers and Potential Intervention Targets for Multiple Sarcopenia-Related Phenotypes
Source: Int J Mol Sci. 2024 Nov 16;25(22):12310. doi: 10.3390/ijms252212310 (PMC11594328; doi:10.3390/ijms252212310)
Supplement: Supplementary file 1 [file ijms-25-12310-s001.zip › Supplementary File S1.pdf]

Supplementary File S1. STROBE-MR checklist of recommended items to address in reports of Mendelian randomization studies[1 2]

Item No.	Section		Checklist item	Page No.	Relevant text from manuscript
1	**TITLE and ABSTRACT**		Indicate Mendelian randomization (MR) as the study's design in the title and/or the abstract if that is a main purpose of the study	2	Mendelian randomization mentioned in abstract.
	**INTRODUCTION**				
2	**Background**		Explain the scientific background and rationale for the reported study. What is the exposure? Is a potential causal relationship between exposure and outcome plausible? Justify why MR is a helpful method to address the study question	3	Introduction section. Line 46-70.
3	**Objectives**		State specific objectives clearly, including pre-specified causal hypotheses (if any). State that MR is a method that, under specific assumptions, intends to estimate causal effects	3	Introduction section. Line 70-74.
	**METHODS**				
4	**Study design and data sources**		Present key elements of the study design early in the article. Consider including a table listing sources of data for all phases of the study. For each data source contributing to the analysis, describe the following:		
		a)	Setting: Describe the study design and the underlying population, if possible. Describe the setting, locations, and relevant dates, including periods of recruitment, exposure, follow-up, and data collection, when available.	4	Method section. Line 263-272;<br>Supplementary Table S1
		b)	Participants: Give the eligibility criteria, and the sources and methods of selection of participants. Report the sample size, and whether any power or sample size calculations were carried out prior to the main analysis	4	Method section. Line 275-295;<br>Supplementary Table S1
		c)	Describe measurement, quality control and selection of genetic variants	5	Method section. Line 298-307.
		d)	For each exposure, outcome, and other relevant variables, describe methods of assessment and diagnostic criteria for diseases	4	Method section. Line 278-284.
		e)	Provide details of ethics committee approval and participant informed consent, if relevant		NA
5	**Assumptions**		Explicitly state the three core IV assumptions for the main analysis (relevance, independence and exclusion restriction) as well assumptions for any additional or sensitivity analysis	5	Method section. Line .308-323.
6	**Statistical methods: main analysis**		Describe statistical methods and statistics used	5	Method section. Metabolome-wide MR analysis<br>Colocalization analysis; Metabolic pathway analysis

		a)	Describe how quantitative variables were handled in the analyses (i.e., scale, units, model)	5	Method section. Line 308-312.
		b)	Describe how genetic variants were handled in the analyses and, if applicable, how their weights were selected	5	Method section. Line 298-316.
		c)	Describe the MR estimator (e.g. two-stage least squares, Wald ratio) and related statistics. Detail the included covariates and, in case of two-sample MR, whether the same covariate set was used for adjustment in the two samples	5	Method section. Line 308-316.
		d)	Explain how missing data were addressed		NA
		e)	If applicable, indicate how multiple testing was addressed	5	Method section. Line 308-316.
7	**Assessment of assumptions**		Describe any methods or prior knowledge used to assess the assumptions or justify their validity	5	Method section. Line 311-316.
8	**Sensitivity analyses and additional analyses**		Describe any sensitivity analyses or additional analyses performed (e.g. comparison of effect estimates from different approaches, independent replication, bias analytic techniques, validation of instruments, simulations)	5	Method section. Line 317-344.
9	**Software and pre-registration**				
		a)	Name statistical software and package(s), including version and settings used	6	Method section. Line 308. Line 325. Line 350.
		b)	State whether the study protocol and details were pre-registered (as well as when and where)		NA
	**RESULTS**				
10	**Descriptive data**				
		a)	Report the numbers of individuals at each stage of included studies and reasons for exclusion. Consider use of a flow diagram		Table 1
		b)	Report summary statistics for phenotypic exposure(s), outcome(s), and other relevant variables (e.g. means, SDs, proportions)		Table 1, Supplementary Table S1
		c)	If the data sources include meta-analyses of previous studies, provide the assessments of heterogeneity across these studies		NA
		d)	For two-sample MR:<br>i. Provide justification of the similarity of the genetic variant-exposure associations between the exposure and outcome samples		NA

			ii. Provide information on the number of individuals who overlap between the exposure and outcome studies		
11	**Main results**				
		a)	Report the associations between genetic variant and exposure, and between genetic variant and outcome, preferably on an interpretable scale		Supplementary Table S2
		b)	Report MR estimates of the relationship between exposure and outcome, and the measures of uncertainty from the MR analysis, on an interpretable scale, such as odds ratio or relative risk per SD difference		Supplementary Tables S4-S7, S9-S16
		c)	If relevant, consider translating estimates of relative risk into absolute risk for a meaningful time period		NA
		d)	Consider plots to visualize results (e.g. forest plot, scatterplot of associations between genetic variants and outcome versus between genetic variants and exposure)		Figures 1-4
12	**Assessment of assumptions**				
		a)	Report the assessment of the validity of the assumptions	7	Results section. Lines 74-75; Table S8
		b)	Report any additional statistics (e.g., assessments of heterogeneity across genetic variants, such as I^2, Q statistic or E-value)	7	Results section. Lines 81-82; Table S8
13	**Sensitivity analyses and additional analyses**				
		a)	Report any sensitivity analyses to assess the robustness of the main results to violations of the assumptions	7	Results section. Lines 102-118.
		b)	Report results from other sensitivity analyses or additional analyses	7	Results section. Lines 95-118.
		c)	Report any assessment of direction of causal relationship (e.g., bidirectional MR)		NA
		d)	When relevant, report and compare with estimates from non-MR analyses		NA
		e)	Consider additional plots to visualize results (e.g., leave-one-out analyses)		NA
	**DISCUSSION**				
14	**Key results**		Summarize key results with reference to study objectives	8	Discussion section. Lines 175-182.
15	**Limitations**		Discuss limitations of the study, taking into account the validity of the IV assumptions, other sources of potential bias, and imprecision. Discuss both direction and magnitude of any potential bias and any efforts to address them	9	Discussion section. Lines 245-261.

16	**Interpretation**				
		a)	Meaning: Give a cautious overall interpretation of results in the context of their limitations and in comparison with other studies	10	Discussion section. Lines 237-261.
		b)	Mechanism: Discuss underlying biological mechanisms that could drive a potential causal relationship between the investigated exposure and the outcome, and whether the gene-environment equivalence assumption is reasonable. Use causal language carefully, clarifying that IV estimates may provide causal effects only under certain assumptions	8	Discussion section. Lines 183-215.
		c)	Clinical relevance: Discuss whether the results have clinical or public policy relevance, and to what extent they inform effect sizes of possible interventions	10	Discussion section. Lines 258-261.
17	**Generalizability**		Discuss the generalizability of the study results (a) to other populations, (b) across other exposure periods/timings, and (c) across other levels of exposure	10	Discussion section. Lines 245-261.
	**OTHER INFORMATION**				
18	**Funding**		Describe sources of funding and the role of funders in the present study and, if applicable, sources of funding for the databases and original study or studies on which the present study is based	11	Funding section.
19	**Data and data sharing**		Provide the data used to perform all analyses or report where and how the data can be accessed, and reference these sources in the article. Provide the statistical code needed to reproduce the results in the article, or report whether the code is publicly accessible and if so, where	11	Availability of data and materials.
20	**Conflicts of Interest**		All authors should declare all potential conflicts of interest	11	Conflict of interest section.

This checklist is copyrighted by the Equator Network under the Creative Commons Attribution 3.0 Unported (CC BY 3.0) license.

1. Skrivankova VW, Richmond RC, Woolf BAR, Yarmolinsky J, Davies NM, Swanson SA, et al. Strengthening the Reporting of Observational Studies in Epidemiology using Mendelian Randomization (STROBE-MR) Statement. JAMA. 2021;under review.

2. Skrivankova VW, Richmond RC, Woolf BAR, Davies NM, Swanson SA, VanderWeele TJ, et al. Strengthening the Reporting of Observational Studies in Epidemiology using Mendelian Randomisation (STROBE-MR): Explanation and Elaboration. BMJ. 2021;375:n2233.
